# Acute Portal Vein Thrombosis during COVID-19 Convalescent Phase

**DOI:** 10.1155/2022/8562978

**Published:** 2022-03-11

**Authors:** Shoja Rahimian, Tushar Pawar, Ian Garrahy, Andrew Rettew

**Affiliations:** Department of Hematology & Oncology, Reading Hospital, West Reading, PA, USA

## Abstract

Acute portal vein thrombosis represents a less common type of venous thromboembolism, even among the prothrombotic complications of coronavirus disease 2019 (COVID-19). Such complications are primarily reported during the active phase of infection. The case here describes acute portal vein thrombosis following resolution of COVID-19 in a 44-year-old male who presented with abdominal pain. Abdominal imaging identified portal and other splanchnic vein thromboses. Studies for hypercoagulable conditions were negative. Polymerase chain reaction was negative for severe acute respiratory syndrome coronavirus-2; however, IgG serology was positive. The case highlights the importance of considering thrombotic complications, particularly splanchnic vein thromboses, in patients with recent COVID-19.

## 1. Background 

Coronavirus disease 2019 (COVID-19) caused by severe acute respiratory syndrome corovirus-2 (SARS-CoV-2) has been associated with hypercoagulability, with recent literature identifying arterial and venous thromboembolism as potential complications [1]. There have also been several case studies that describe portal vein thrombosis (PVT) complicating COVID-19, most of which occurred at the time of acute respiratory illness [2–11]. We present a case of acute portal vein thrombosis in the convalescent phase as identified by the presence of SARS-CoV-2 IgG antibodies.

## 2. Case Presentation

A 44-year-old Hispanic male with no significant past medical history presented to the emergency department with severe intermittent abdominal pain of four-week duration with acute worsening prior over 3-4 days. He had experienced fever, nausea, anosmia, and dry cough four weeks prior at which time he self-quarantined for 14 days, given that concern for COVID-19. SARS-CoV-2 testing was not performed at that time. His symptoms resolved within one week of isolation. On arrival, he was afebrile (38.2°C), tachycardic (115 beats per minute), and with normal blood pressure (124/74 mmHg). Oxygen saturation was 94% on room air. He endorsed worsening generalized abdominal pain, but denied nausea, vomiting, or diarrhea. Comprehensive metabolic panel was significant for elevated AST of 44 (range: 13–39 IU/L) and ALT of 172 (range: 7–52 IU/L). CBC was unremarkable. Acute hepatitis panel and serum lipase were negative, while AFP tumor marker was undetectable. Other pertinent labs included elevated CRP, 8.60 (range: <1 mg/dL); ESR, 18 (range: 0–15 mm/hr); ferritin, 385 (range: 24–336 ng/ml); and D-dimer 14,540 (normal <500 ug/L). Given his recent symptoms, a nasal swab SARS-CoV-2 RNA polymerase chain reaction (PCR) was ordered which was negative.

**Figure 1:**
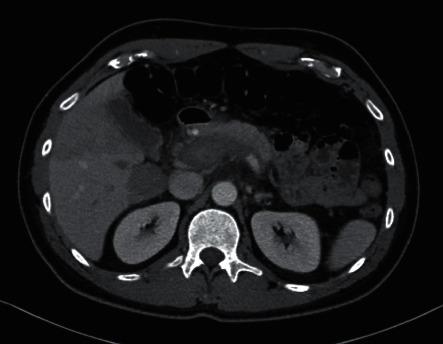
CT scan of abdomen/pelvis showing portal vein thrombosis.

**Figure 2:**
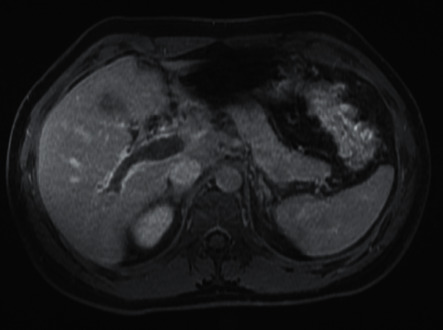
MRI of abdomen and pelvis with portal vein thrombosis.

A computed tomographic (CT) scan of abdomen with contrast (Figure 1) demonstrated dilation with heterogeneous density involving the portal and splenic veins associated with surrounding inflammation concerning for portal vein thrombosis. Subsequent magnetic resonance imaging of the abdomen/pelvis (Figure 2) revealed findings consistent with extensive thrombosis of the portal vein extending from the superior mesenteric and splenic veins through to the intrahepatic branches most pronounced within the right hepatic lobe. Given his extensive PVT, absence of personal or family history of thrombosis, and no evidence for underlying malignancy, further workup for hypercoagulable state was commenced. Myeloproliferative neoplasm panel was negative for JAK2 V617F, JAK2 exon 12–14, CALR, and MPL mutations. High sensitivity flow cytometry for paroxysmal nocturnal hemoglobinuria was also negative. Other pertinent negatives included factor V Leiden mutation, prothrombin gene mutation, anticardiolipin antibodies, beta-2 glycoprotein antibodies, and lupus anticoagulant.

Given the negative workup and considering his prior illness suspicious for COVID-19, a SARS-CoV-2 IgG antibody test was ordered which returned positive, signifying prior disease in this patient who had not been vaccinated. He was initially started on enoxaparin 1 mg/kg twice daily and then transitioned to rivaroxaban at discharge. He completed 6 months of oral anticoagulation with complete resolution of thrombosis on posttreatment CT abdomen with no plan for further anticoagulation given the provoked nature of the thrombosis in the setting of COVID-19.

## 3. Discussion

The clinical presentation of COVID-19 infection is variable, ranging from a flu-like syndrome with mild respiratory symptoms to viral pneumonia with acute respiratory failure, multiorgan dysfunction, and death. Studies and clinical experience identify that patients with COVID-19 infection have an acquired hypercoagulable state with predisposition to thrombotic events [12].

Case reports have documented a tendency of SARS-CoV-2 to induce a hypercoagulable state, including the formation of in situ pulmonary embolism. Several cases of PVT associated with COVID-19 have been reported in the literature to date. The prevalence of PVT in the general population is rare at around 1% as evidenced in a Swedish study where 23,796 consecutive autopsies demonstrated presence of PVT in 254 patients. 28% of these cases were associated with liver cirrhosis, 44% primary and secondary hepato-biliary malignancies, 10% abdominal infections, 3% in myeloproliferative neoplasms, and 14% idiopathic [13]. PVT with COVID-19 represents a less common association, but one that requires further review and acknowledgement. Our case represents the ninth such reported COVID-19-associated PVT [2–9].

An in-depth review of the other eight case reports showed that most PVT occurred while the patients were hospitalized. Similar to the case described by Franco-Moreno et al., our case is unique in which the portal vein thrombosis occurred after resolution of his respiratory symptoms several weeks prior. PCR testing was negative, and the diagnosis of COVID-19-associated portal vein thrombosis was made using antibody testing in an individual who had not been previously vaccinated.

The highly inflammatory potential of SARS-CoV-2 is thought to be related to its prothrombotic pathogenicity. It has been shown that the virus binds to the transmembrane angiotensin-converting enzyme 2 (ACE2) protein to enter type II pneumocytes, macrophages, and other cell types. In humans, an abundant expression of ACE2 receptors on endothelial cells enhances their vulnerability to SARS-CoV-2 binding, membrane fusion, and viral entry causing vascular injury [14–16]. SARS-CoV-2 infection of the host can affect the ACE/ACE2 ratio, leading to downregulation of ACE2 [17]. An increase in the ACE/ACE2 ratio is profibrotic and proinflammatory. Most viral infections lead to host cell death leading to the local intense inflammatory response that may become systemic because of the potent proinflammatory cytokines, such as interleukin-1*β* and interleukin-18 [18]. The ACE2-facilitated inflammatory response can lead to development of hepatic portal venous gas in COVID-19 which is a possible finding in the patients with abdominal pain [19].

Platelets represent the interplay between hemostasis and the immune system. The potent local and systemic cytokine production leads to platelet activation and interaction with neutrophils. Leukocyte activation, specifically neutrophils, through various vascular and platelet pathways, may promote neutrophil extracellular trap (NET) formation. NETs are large, extracellular, weblike structures composed of cytosolic and granule proteins assembled on a scaffold of decondensed chromatin [20]. NETs are an ideal foundation for binding activated platelets, erythrocytes, and leukocytes, and activating factor XI, and generating thrombin for fibrin production. Panigada et al. assessed several coagulation parameters in patients with COVID-19 [21]. Using whole blood thromboelastography, the authors identified hypercoagulability features, such as a decrease in time to fibrin formation, a decrease in time to clot formation, and increase in clot strength. Using thromboelastographic analysis, other authors found low lysis at 30 minutes, which is suggestive of fibrinolysis shutdown. The excess of fibrin deposition and fibrinolysis shutdown leads to intravascular thrombosis and, finally, to clinical thromboembolic complications.

## 4. Conclusion

Acute thrombotic complication associated with COVID-19 has been reported in the literature with most cases occurring within the COVID-19 hospitalization and consists mainly of deep vein thrombosis and pulmonary embolism. This case gives an insight into rare thrombotic complication of PVT occurring in relatively mild COVID-19, and a few weeks after resolution of his infection-associated symptoms. This case highlights the importance of considering thrombotic complications in patients with ongoing or recent COVID-19 and thus a low threshold for imaging studies and treatment as indicated.
